# Interventions to Promote Healthy Eating, Physical Activity and Smoking in Low-Income Groups: a Systematic Review with Meta-Analysis of Behavior Change Techniques and Delivery/Context

**DOI:** 10.1007/s12529-018-9734-z

**Published:** 2018-07-12

**Authors:** Eleanor R. Bull, Nicola McCleary, Xinru Li, Stephan U. Dombrowski, Elise Dusseldorp, Marie Johnston

**Affiliations:** 1 Manchester Metropolitan University, Manchester, UK; 2 University of Manchester, Manchester, UK; 3 Ottawa Hospital Research Institute, Ottawa, Canada; 4 Mathematical Institute, Leiden University, Leiden, the Netherlands; 5 University of Stirling, Stirling, UK; 6 Institute of Psychology, Leiden University, Leiden, the Netherlands; 7 University of Aberdeen, Aberdeen, UK

**Keywords:** Behavior change techniques, Healthy eating, Physical activity, Smoking cessation, Low-income populations, Meta-analysis

## Abstract

**Purpose:**

Healthy eating, physical activity and smoking interventions for low-income groups may have small, positive effects. Identifying effective intervention components could guide intervention development. This study investigated which content and delivery components of interventions were associated with increased healthy behavior in randomised controlled trials (RCTs) for low-income adults.

**Method:**

Data from a review showing intervention effects in 35 RCTs containing 45 interventions with 17,000 participants were analysed to assess associations with behavior change techniques (BCTs) and delivery/context components from the template for intervention description and replication (TIDieR) checklist. The associations of 46 BCTs and 14 delivery/context components with behavior change (measures of healthy eating, physical activity and smoking cessation) were examined using random effects subgroup meta-analyses. Synergistic effects of components were examined using classification and regression trees (meta-CART) analyses based on both fixed and random effects assumptions.

**Results:**

For healthy eating, self-monitoring, delivery through personal contact, and targeting multiple behaviors were associated with increased effectiveness. Providing feedback, information about emotional consequences, or using prompts and cues were associated with reduced effectiveness. In synergistic analyses, interventions were most effective without feedback, or with self-monitoring excluding feedback. More effective physical activity interventions included behavioral practice/rehearsal or instruction, focussed solely on physical activity or took place in home/community settings. Information about antecedents was associated with reduced effectiveness. In synergistic analyses, interventions were most effective in home/community settings with instruction. No associations were identified for smoking.

**Conclusion:**

This study identified BCTs and delivery/context components, individually and synergistically, linked to increased and reduced effectiveness of healthy eating and physical activity interventions. The identified components should be subject to further experimental study to help inform the development effective behavior change interventions for low-income groups to reduce health inequalities.

**Electronic supplementary material:**

The online version of this article (10.1007/s12529-018-9734-z) contains supplementary material, which is available to authorized users.

## Background

People of lower socioeconomic status are less likely to eat healthily [1] or be physically active and [2] more likely to smoke [3] compared to those of higher socioeconomic status. These behaviors may be mediators of the well-established link between social position and morbidity and mortality outcomes [4–6]. Amongst many socioeconomic indicators including education levels, measures of job status and access to healthcare, personal or household income is a direct economic indicator which is strongly positively correlated with health outcomes [1]. In trials of interventions for the general population, people with a lower income may experience poorer behavior change outcomes than more affluent participants potentially leading to intervention-generated inequalities [7–10]. Targeting health promotion efforts at people facing deprivation may prevent ill health and contribute towards reducing health inequalities [6].

A previous review of interventions targeted at low-income participants found that approximately half were effective [11]. Furthermore, a more recent systematic review with meta-analysis found positive, but small and variable effects on healthy eating (standardised mean difference (SMD) 0.22, I^2^ = 48%), physical activity (SMD 0.21, I^2^ = 76%) and smoking (relative risk (RR) 1.59, I^2^ = 40%), smaller than other similar interventions with participants of mixed income [12]. Initial explorations of heterogeneity were conducted in that review but associations between specific intervention components with variation in intervention effectiveness were not examined. Understanding this variability, including identifying potentially underutilised effective components with these groups, is important when health inequalities continue to widen [10].

Behavioral medicine researchers have recently developed several frameworks and tools to help us accurately and comprehensively describe intervention components and accumulate evidence of ‘what works’. The template for intervention description and replication (TIDieR) checklist specifies 12 elements of healthcare interventions which study authors should report, including aspects of delivery and context [13]. These include describing ‘how’ they took place, i.e. the mode of delivery (e.g. face-to-face, telephone), ‘where’, or the setting (e.g. at home, in a school, or in a health facility) and ‘what’ content was delivered. For further characterising this, researchers have developed a shared language known as the behavior change technique taxonomy (BCTTv1) [14], including 93 active ingredients of behavior change interventions called behavior change techniques (BCTs). Better understanding the content, delivery and context of existing behavior change interventions for low-income groups and exploring which seem effective and ineffective could prove timely and useful.

Recently, a promising new statistical method called Meta-CART has been developed to help analyse the effectiveness of combinations of BCTs and other intervention features [15, 16]. Most health behavior change interventions are complex [17] containing many BCTs and delivery/context components which can amplify or attenuate each other’s effect [15]. It has been argued that analyses must consider or control for this ‘co-occurrence of methods’ to advance behavior change science [18]. For instance, healthy eating interventions could involve combinations of goal setting, self-monitoring of behavior, and/or practical social support delivered by a health coach on the telephone or via a mailed leaflet. Traditional moderator analysis only examines the effect of each moderator individually, whereas meta-CART can use subgroup meta-analysis to identify interactions between moderators across interventions, such as to explore whether goal setting may be best delivered on the telephone or via a leaflet.

## Aim and Objectives

This study aimed to conduct a new analysis of data from a previously published systematic review of health promotion interventions for low-income groups [12], applying behavioral science frameworks and new statistical methods to understand more about their effectiveness. While the previous paper found interventions to have small, positive effects, the current paper investigates which critical features of intervention content and delivery may contribute to their effectiveness. The association between a range of intervention components, individually and in combination, with variability in intervention effect sizes was examined. There were two specific objectives:

1. To explore which individual BCTs and delivery/context features such as those from the TIDieR checklist are associated with effectiveness by applying moderator analyses.
2. To explore synergistic effects between BCTs and delivery/context components and identify combinations associated with effectiveness by applying the new method meta-CART.

## Methods

The study was registered in the PROSPERO database (CRD42015017468) and completed as per protocol. We applied moderator analyses to the data from a previously published systematic review with meta-analysis. For clarity, the original review’s eligibility criteria, search strategy and data collection processes are summarised below in this section, but further detail can be found in the published paper (http://bmjopen.bmj.com/content/4/11/e006046) [12].

## Original Review Method Summary

The original review by Bull et al. [12] included studies meeting the following inclusion criteria: (i) population: currently healthy adults described in the study as ‘low-income’; (ii) interventions: aiming to change healthy eating, physical activity and/or smoking behavior in any combination; (iii) study design: RCTs or Cluster RCTs, with no limits on control condition design; (iv) outcomes: behavioral outcomes relevant to healthy eating, physical activity or smoking (e.g. self-reported portions of fruit per day, accelerometer-measured steps walked per week, or self-reported abstinence from smoking for seven consecutive days); (v) date: primary search carried out January 2006 to July 2014; (vi) language: English.

Bull et al. [12] searched eight databases for studies with terms relating to low-income groups, terms for healthy eating, physical activity and smoking behaviors, and terms relating to interventions and health programs. In addition, in Bull et al. [12] studies published between 1995 and 2006 were identified from another previously published review without meta-analysis on the topic [11] rather than through a primary search and screened against the inclusion criteria listed above, since Michie et al. [11] used similar but broader search criteria which should have included all the relevant articles. Finally, in addition to these searches, Bull et al. [12] checked each included study’s bibliography for potentially relevant articles to screen.

In Bull et al. [12], three authors screened titles and abstracts; one author screened full texts. In both stages, double screening of a random 10% yielded high inter-rater reliability. Data were collected using a piloted data extraction form based on Davidson et al. [19]. Three authors jointly extracted design, methods and results data. Proportions were extracted for dichotomous smoking outcomes; means and standard deviations were extracted for continuous healthy eating and physical activity outcomes. Where there was a choice, the outcome extracted was the primary measure specified by authors measured as objectively as possible, adjusted for baseline if the authors had thought this necessary. Risk of bias in individual studies was assessed based on standard criteria adapted from Avenell et al. [20] and publication bias inspected visually using a funnel plot, reported in Bull et al. [12].

## Current Review Methods

**Fig. 1:**
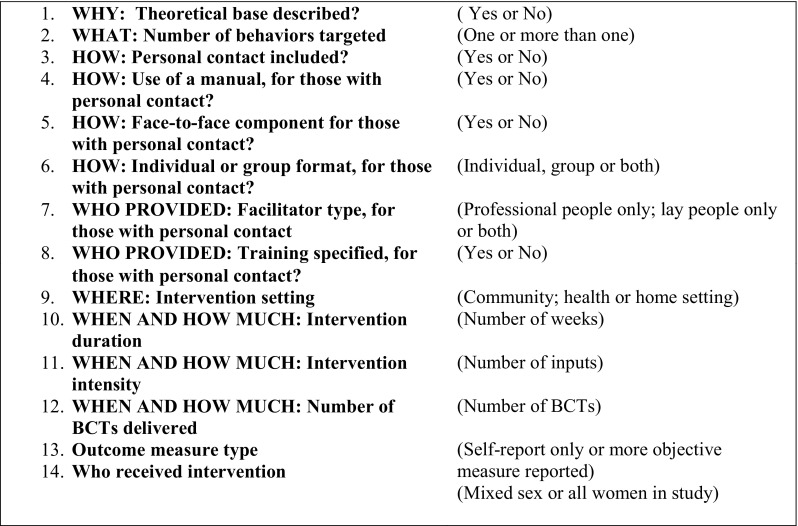
Fourteen delivery/context components based on the TIDieR checklist

In the new analysis, content and delivery/context component data were extracted from intervention descriptions in studies. Two authors jointly coded 14 components of each intervention, including 12 components based on the TIDieR checklist [13] with the addition of ‘WHO RECEIVED’ the intervention and the outcome measure type (see Fig. 1 for the list of 14 components). A trained coder extracted each intervention’s BCTs using BCTTv1 [14]. Two expert coders extracted the BCTs in a random subset of 16 studies’ intervention descriptions to assess coding reliability. Prevalence and bias-adjusted kappa (PABAK) [21] was 0.87 and 0.83 respectively, suggesting high inter-rater agreement [22]. Published online supplementary materials were used where available and the corresponding author was contacted in the case of missing data.

## Statistical Analysis

### Moderator Analysis

For continuous healthy eating and physical activity outcomes, standardised mean differences (SMDs) were calculated using Hedges *g*. For dichotomous smoking outcomes, we calculated relative risk (RR) of smoking abstinence and applied the Cochran-Mantel-Haenszel test [23]. To minimise chance impact of single trials, we only examined BCTs and intervention delivery components identified as present or absent in at least three interventions as potential moderators, following Dombrowski et al. [24]. Each BCT and categorical delivery/context variable was examined testing subgroup differences using a mixed effects model (i.e., a random effects model for the within-subgroup effect sizes, and a fixed effect model for testing the between-subgroups heterogeneity, as recommended by Borenstein et al.) [25]. Three continuous intervention components (intervention intensity, duration and number of BCTs) were analysed using meta-regression. We used comprehensive meta-analysis (v.2) for these analyses.

### Meta-CART Analysis

To explore interaction effects and identify effective combinations of BCTs and delivery/context components, meta-CART was applied. Meta-CART is a tree-based method that combines the machine learning technique CART (Classification And Regression Trees) with meta-analysis [26]. Meta-CART uses study effect sizes (e.g. the study SMDs for healthy eating) as outcome variables, and potential moderators as predictor variables (e.g. BCTs). The method divides the study effect sizes into homogeneous subgroups of interventions based on influential moderators. The result of a meta-CART analysis is a tree, and the leaves (the end nodes) of the tree are subgroups of studies with similar combinations of moderators. In each subgroup, a pooled effect size is computed (i.e. a weighted average effect size). The pooled effect sizes of these subgroups are as different as possible since meta-CART maximises the between-subgroups heterogeneity (i.e. *Q*-statistic) [16]. At each split of the tree, the meta-CART algorithm searches for the moderator (i.e. a BCT/delivery or context variable) that maximises the between-subgroups differences. Initially, a large tree is grown with as many splits as possible. The best tree size is selected by ‘pruning’, removing the spurious splits of the tree based on the cross-validation error [26]. The final tree usually involves a smaller number of splits, for example a tree with two moderators (e.g. Fig. 3). The tree represents synergistic effects between the moderators involved in the splits of the tree. To test whether the synergistic effects significantly explain the heterogeneity between interventions, a new moderator variable is computed with categories referring to the end nodes (i.e. subgroups) of the tree, with each study belonging to one specific subgroup. Finally, a standard subgroup meta-analysis is performed using the new moderator variable to investigate whether the subgroup membership accounts for the heterogeneity in the study effect sizes.

Meta-CART analyses were performed using both random effects (RE) and fixed effect (FE) approaches to explore effective combination(s) of moderators. Both methods have advantages: RE methods are more conservative, maximising control of type 1 error [16] whereas the more liberal FE method favours power, so applying both was seen as appropriate offering complementary information in this exploratory study. The RE meta-CART takes into account the residual heterogeneity unexplained by the individual BCTs and delivery/context components, whereas FE meta-CART assumes that the heterogeneity in study effect sizes is fully explained by the moderators. For both approaches, initial trees are grown with nodes of at least two interventions, before pruning. We used the half-standard-error pruning rule for the RE meta-CART analyses (i.e., selecting the smallest tree that has a cross-validation error within the minimum cross-validation error plus half times the standard error) [16]. The FE meta-CART used the minimum cross-validation error pruning rule, selecting the tree with minimum cross-validation error to increase power. Since the number of interventions was relatively small in our meta-analytic data sets, we performed leave-one-out cross-validation for pruning, although for larger data sets a tenfold cross-validation is generally recommended.

In total, six meta-CART analyses were performed: FE and RE analyses for healthy eating, physical activity and smoking interventions. Due to the relatively small number of interventions, for the meta-CART analyses we included the BCTs and delivery/context components which had shown significant effects in the univariate moderator analysis. All meta-CART analyses were performed using R (v.3.3.2).

## Results

### Original Review Study Selection and Characteristics

In the original review [12], 2569 titles and abstracts and 133 full texts were screened. Thirty-five trials were included with 17,000 adult participants with a low income. Thirty trials were conducted in the USA; three in the UK; one in Australia and Chile. Eleven studies recruited participants with a specific ethnic background (African-American, Latina or Chinese and Korean). The majority of participants were women (72.4%) living in the USA (77.2%) with a mean age of 38.6 years. The 35 studies contained 45 interventions: some studies targeted multiple behaviors or tested multiple interventions. In all, there were 16 interventions targeted at healthy eating; 12 at physical activity; 17 at smoking.

### Current Review Study Characteristics

Of the 93 BCTs in the taxonomy and the 14 delivery/context components, 46 BCTs and all 14 delivery/context components were identified from the 45 published intervention descriptions. Each intervention contained between 2 and 20 BCTs (mean per intervention 6.62). Of the three delivery/context components which were continuous moderators (WHEN AND HOW MUCH: Intervention duration, Intervention intensity and Number of BCTs delivered; Fig. 1), none were associated with effectiveness for any behavior, so here we present results for the categorical BCTs and 11 remaining delivery/context components only. Amongst these, four delivery/context components were not applicable to interventions without personal contact (use of a facilitator delivery manual; facilitator type; facilitator training; group or individual format) so were only examined for interventions with personal contact. In total, 23 BCTs and seven delivery/context components were present or absent in at least three interventions (see methods statistical analysis section) and analysed as potential moderators.

### Healthy Eating: Individual Moderator Analysis

**Fig. 2:**
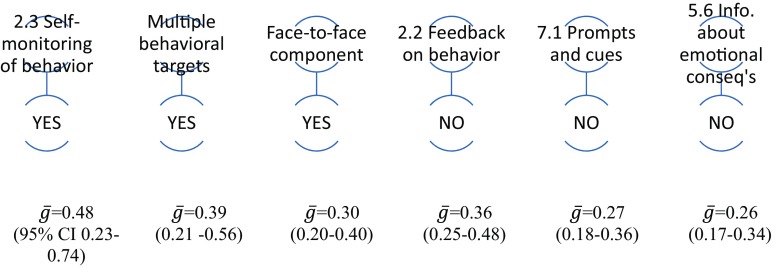
Diagram representing univariate moderator analyses for healthy eating. BCTs are presented with their original labels and number from BCTv1 [14]. In Figs. 2, 3, 4, 5, and 6, ğ represents effect size and 95% CIs statistical significance. Figure 2 indicates that healthy eating interventions were significantly more effective where they *did* include the BCT 2.3 Self-monitoring of behavior, or if there were multiple behavioral targets or a face-to-face component, or *did not* include BCTs 2.2 Feedback on behavior, 7.1 Prompts and cues or 5.6 Information about emotional consequences

Sixteen BCTs and seven delivery/context components could be analysed within the 16 healthy eating interventions. Interventions including the BCT *2.3 Self-monitoring of behavior* were associated with more healthy eating, while those with the BCTs *2.2 Feedback on behavior*, *7.1 Prompts and cues* or *5.6 Information about emotional consequences,* were associated with less healthy eating (Supplementary Table 1)*.* Amongst delivery/context components, including a face-to-face component (rather than remote contact, e.g. by telephone, or no personal contact) and a multi-behavioral focus (aiming to change both healthy eating and another behavior) were also associated with increased effectiveness (Supplementary Table 1). Figure 2 displays the statistically significant findings of the individual moderator analysis visually.

### Healthy Eating: Meta-CART Analysis of Synergistic Effects

**Fig. 3:**
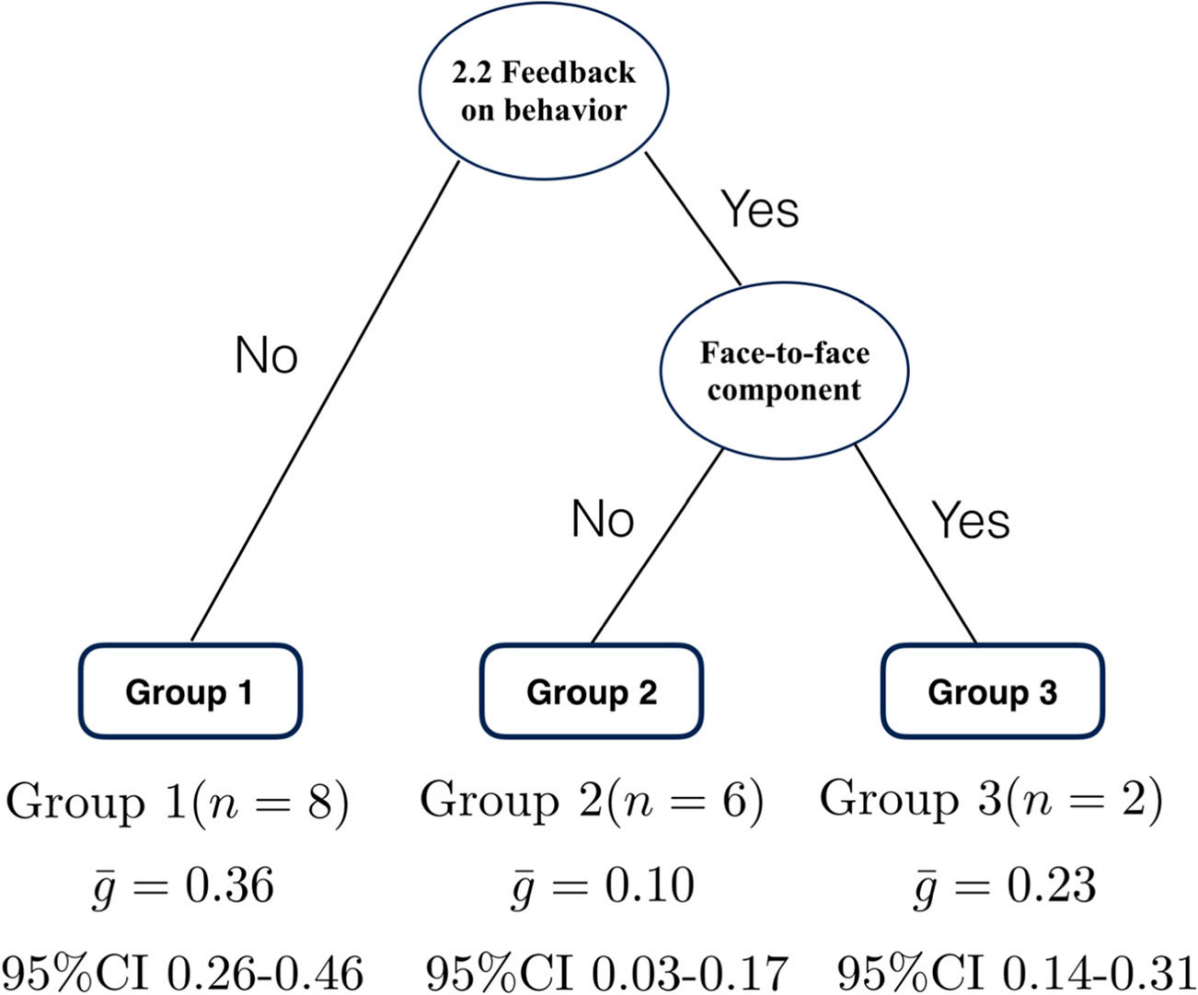
Results from random effects meta-CART meta-analysis for healthy eating *(k* = 16). Figure 3 indicates random effects meta-CART analysis of effective combinations of the four BCTs and two delivery/context components identified as individually significant moderators in Fig. 2. Healthy eating interventions were more effective if they did not include the BCT 2.2 Feedback on behavior, but if they did, then those with a Face-to-face delivery component were more effective than those without

Meta-CART was conducted to identify effective combinations of the four BCTs and two delivery/context components identified above in individual moderator analyses (Supplementary Table 1). The tree that resulted from the RE meta-CART analysis represented a synergistic effect between *2.2 Feedback on behavior* and face-to-face component (Fig. 3). The interventions that excluded *2.2 Feedback on behavior* showed the highest pooled effect size (i.e., $\overline{g}=$0.36, 95% CI 0.26–0.46). When *2.2 Feedback on behavior* was included, the interventions that also included a face-to-face component had a larger pooled effect size ($\overline{g}=$0.23, 95% CI 0.14–0.31) than the interventions without ($\overline{g}=$0.10, 95% CI 0.03–0.17). In the mixed effects subgroup meta-analysis, subgroups were significantly different from each other (between-subgroups *Q*-statistic = 17.49, *p* = .002).

**Fig. 4:**
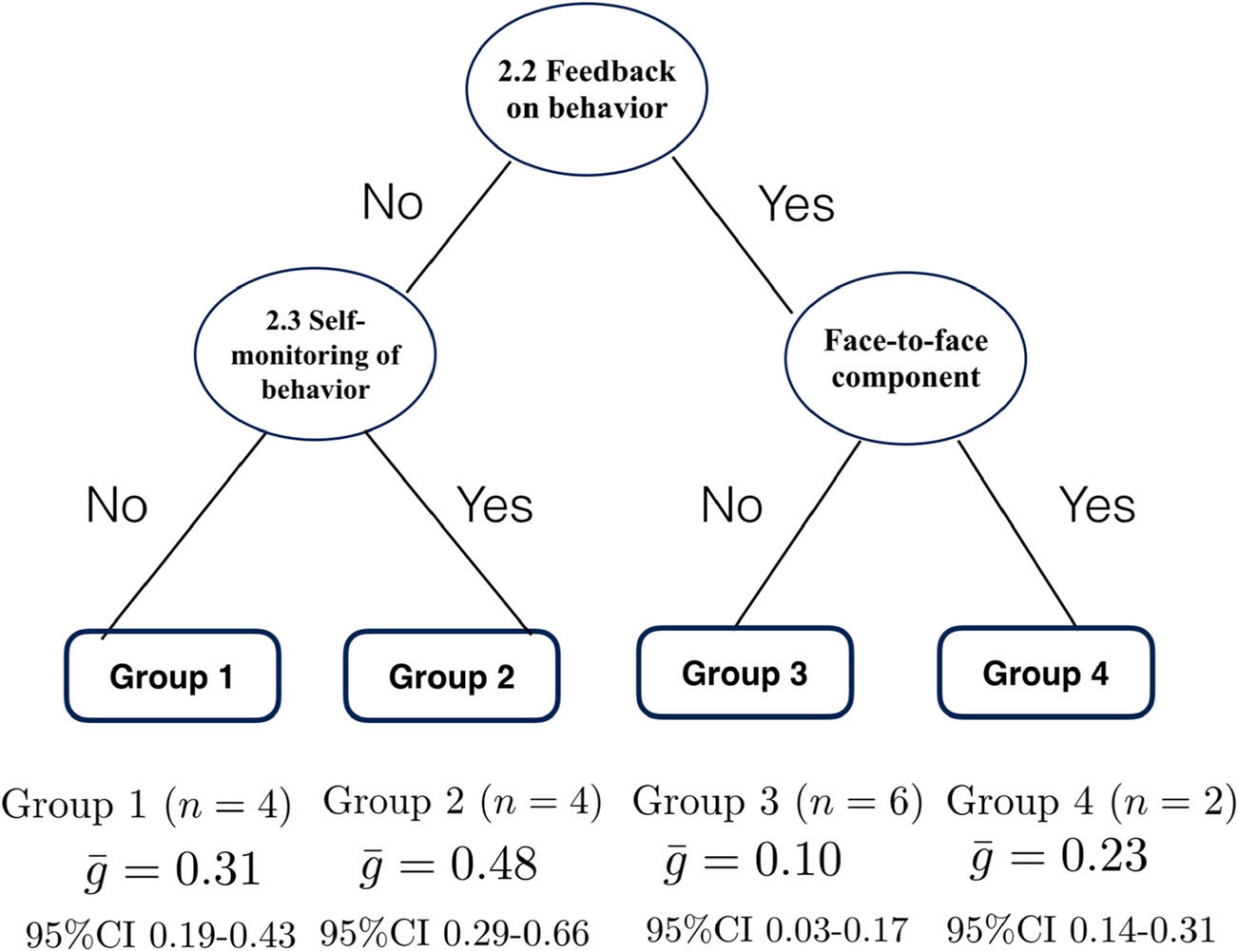
Results from fixed effects meta-CART meta-analysis for healthy eating (*k* = 16). Figure 4 indicates fixed effects meta-CART analysis of effective combinations of the four BCTs and two delivery/context components identified as individually significant moderators in Fig. 2. Results were similar to Fig. 3, but also indicated that interventions excluding the BCT 2.2 Feedback on behavior but including 2.3 Self-monitoring of behavior were most effective

Compared with the RE meta-CART analysis results, the tree resulting from the FE meta-CART analysis included one additional split: a synergistic effect between *2.2 Feedback on behavior* and *2.3 Self-monitoring of behavior* (Fig. 4). The interventions that used *2.3 Self-monitoring of behavior* but excluded *2.2 Feedback on behavior* were most effective ($\overline{g}=$0.48, 95% CI 0.29–0.66). The interventions using both *2.3 Self-monitoring of behavior* and *2.2 Feedback on behavior* were least effective ($\overline{g}$= 0.31, 95% CI 0.19–0.43). The synergistic effect between *2.2 Feedback on behavior* and face-to-face component was the same as the RE meta-CART result. In the FE subgroup meta-analysis, again subgroups were significantly different from each other (between-subgroups *Q*-statistic = 19.68, *p* = .002).

### Physical Activity: Individual Moderator Analysis

**Fig. 5:**
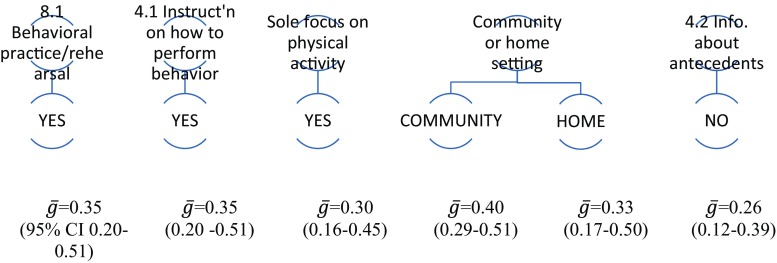
Diagram representing univariate moderator analyses for physical activity. Figure 5 indicates that physical activity interventions were significantly more effective where they *did* include the BCTs 8.1 Behavioral practice/rehearsal or 4.1 Instruction on how to perform the behavior, or had a sole focus on physical activity, or were delivered in a community or home (rather than health) setting, or *did not* include the BCT 4.2 Information about antecedents

Fourteen BCTs and six delivery/context components could be analysed within the 12 physical activity interventions. Interventions including *8.1 Behavioral practice / rehearsal,* or *4.1 Instruction on how to perform behavior*) were associated with increased physical activity; interventions including the BCT *4.2 Information about antecedents* with less physical activity. In addition, intervention delivery in a community or home setting (rather than in a health setting) and a sole focus on physical activity was associated with greater effectiveness (Supplementary Table 2). Figure 5 displays the statistically significant findings visually of the individual moderator analysis.

### Physical Activity: Meta-CART Analysis of Synergistic Effects

Meta-CART was conducted to identify effective combinations of the three BCTs and two delivery/context components identified as individual moderators above (Supplementary Table 2). RE meta-CART resulted in a tree with only one node: the root node: no combination of BCTs or delivery/context components was able to explain the heterogeneity in the effect sizes.

**Fig. 6:**
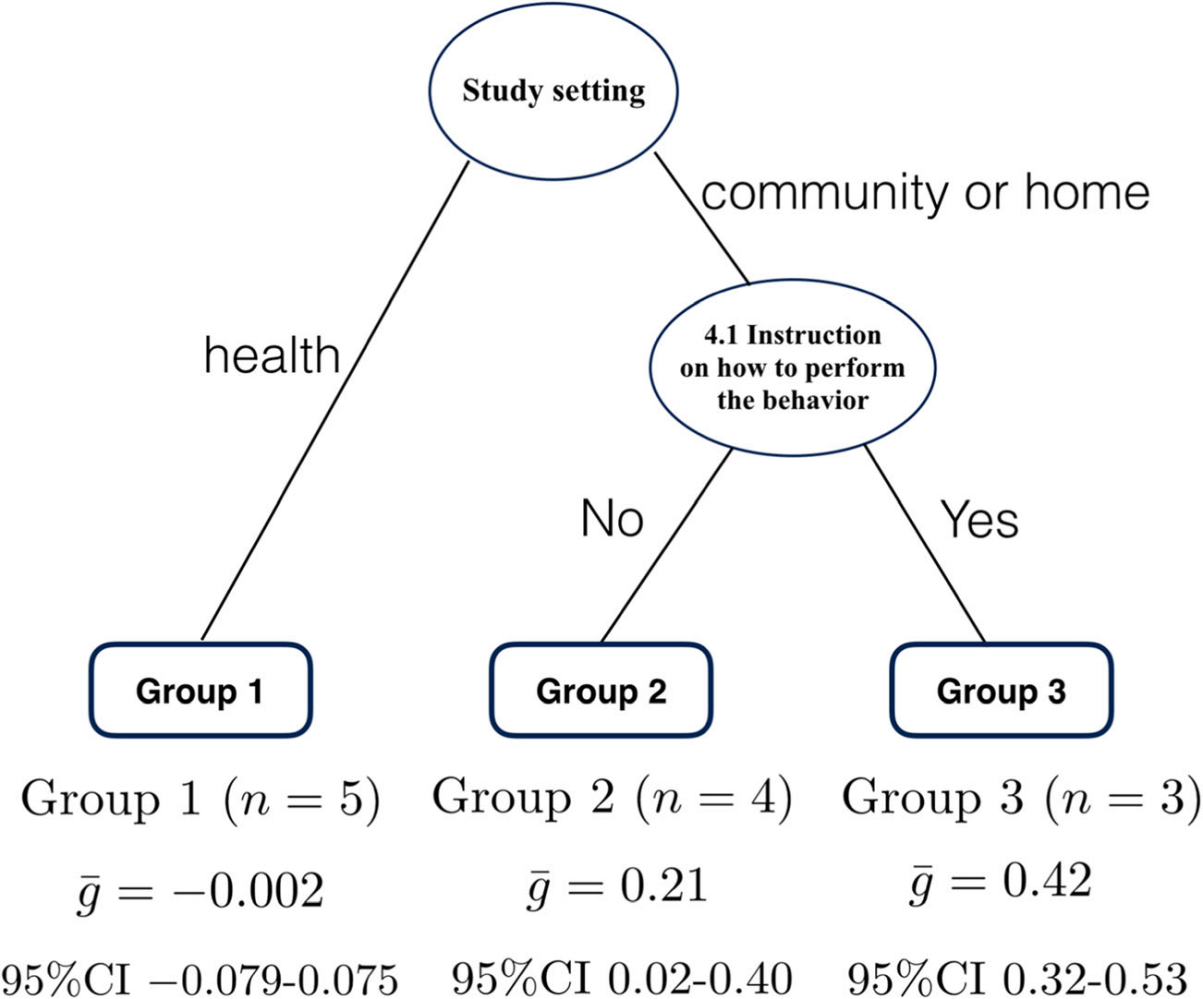
Results from fixed effects meta-CART meta-analysis for physical activity (*k* = 12). Figure 6 indicates fixed effects meta-CART analysis of effective combinations of the three BCTs and two delivery/context components identified as individually significant moderators in Fig. 5. Physical activity interventions were more effective if they were delivered in a community setting or at home and included the BCT 4.1 Instruction on how to perform the behavior, and were least effective if delivered in a health setting

The tree resulting from the FE meta-CART analysis represented a synergistic effect between *4.1 Instruction on how to perform behavior* and study setting. The interventions delivered in health settings had the lowest pooled effect size ($\overline{g}$ = −0.002, 95% CI -0.079-0.075). Interventions delivered in community or home settings which included *4.1 Instruction on how to perform behavior* had the highest pooled effect size ($\overline{g}$ = 0.42, 95% CI 0.32–0.53). Interventions delivered in community or home settings but not including *4.1 Instruction on how to perform behavior* had a pooled effect size in the middle of the other subgroups ($\overline{g}$ = 0.21, 95% CI 0.02–0.40). All of the interventions delivered in community or home settings applied either both *4.1 Instruction on how to perform behavior* and *8.1 Behavioral practice /rehearsal* or neither, none included just one. In the FE subgroup meta-analysis, subgroups were significantly different from each other (between-subgroups *Q*-statistic = 43.18, *p* < .001). Figure 6 displays this visually.

### Smoking: Individual Moderator Analysis

Eleven BCTs and five delivery/context components were analysed within the 17 smoking interventions, none of which were statistically associated with smoking intervention effectiveness (Supplementary Table 3).

### Smoking: Meta-CART Analysis of Synergistic Effects

Both RE meta-CART and FE meta-CART detected no effective combination of BCTs or delivery/context components that could explain the heterogeneity in the effect sizes.

Table 1, summarises the individual BCTs and delivery/context components associated with increased or decreased effectiveness in healthy eating and physical activity with examples from the interventions included in the review.

**Table 1:** Definitions and examples of BCTs and delivery/context components associated with increased or decreased effectiveness

7	BCT or delivery/context component	Definition *BCT numbers, labels and definitions are taken from BCTV1 [14]	Example from interventions included in the review
Increased effectiveness DIET	2.3 Self-monitoring of behaviour*	Establish a method for the person to monitor and record their behaviour(s) as part of a behaviour change strategy*	In Keyserling et al. [27], participants recorded daily fruit and vegetables consumed each day using a diary to help them increase this
	HOW: Face-to-face component included (yes)	For studies with personal contact, whether or not this personal contact was conducted face-to-face (instead of e.g. over telephone)	Emmons et al. [28] included a counselling session in-person with a health advisor using motivational interviewing approaches to help support them to eat more healthily
	Number of behaviours targeted (multiple focus)	Whether the study aimed to change one behaviour (e.g. diet only) or multiple behaviours (e.g. diet and physical activity)	Jackson et al. [29] focused on participants making healthy changes to both diet and physical activity
Decreased effectiveness DIET	2.2 Feedback on behaviour*	Monitor and provide informative or evaluative feedback on performance of the behaviour (e.g. form, frequency, duration, intensity)*	Elder et al. [36] provided individualised written feedback to participants from an earlier assessment e.g. their current diet compared to national norms
	7.1 Prompts and cues*	Introduce or define environmental or social stimulus with the purpose of prompting or cueing the behaviour. The prompt or cue would normally occur at the time or place of performance*	Participants in Tessaro et al.’s study [30] received a portion magnet and wheel to put in their kitchen to remind them of healthy portion sizes
	5.6 Information about emotional consequences*	Provide information (e.g. written, verbal, visual) about emotional consequences of performing the behaviour*	Gans et al. [37] included a video with testimonials from members of the target audience, who emphasised that eating healthier helps in feeling good about yourself and feeling better
Increased effectiveness PHYSICAL ACTIVITY	8.1 Behavioral practice/ rehearsal*	Prompt practice or rehearsal of the performance of the behaviour one or more times in a context or at a time when the performance may not be necessary, in order to increase habit and skill*	Marcus et al. [31] included tailored written mailings which advised participants, for example, to make time for a ten minute walk one or two times each week, to help them build confidence that they can make exercise part of their weekly routine
	4.1 Instruction on how to perform a behavior*	Advise or agree on how to perform the behaviour (includes ‘Skills training’)*	Dangour et al.’s physical activity program for older adults [32] included physical activity group training sessions where trained instructions guided participants in how to conduct activities e.g. warming up, chair stands, modified squats and arm pull-ups with rubber bands.
	WHERE: Study setting (community or at home, not in health setting)	Whether the study was set in the community, a health setting or at participants’ home	Olvera et al.’s 12 week exercise program [33] took place in community centres in the park, park playgrounds and grocery stores, as well as at school facilities, e.g. the school gym, playground or cafeteria
	Number of behaviors targeted (single focus)	Whether the study aimed to change one behavior (e.g. physical activity only) or multiple behaviours (e.g. diet and physical activity)	Dutton et al.’s intervention [34] focused solely on increasing women’s physical activity
Decreased effectiveness PHYSICAL ACTIVITY	4.2 Information about antecedents*	Provide information about antecedents (e.g. social and environmental situations and events, emotions, cognitions) that reliably predict performance of the behaviour*	Chang et al. [35] provided examples of triggers relating to eating and being active in the environment as part of their behaviour change intervention

## Discussion

In this study, we explored active components of interventions (BCTs) and the context and methods of delivery associated with effectiveness in health behavior change interventions for low-income adults, applying both individual moderator analyses and meta-CART to explore combinations of components. The content, context and delivery of effective interventions appeared different for healthy eating and physical activity behaviors. For healthy eating behavior, individual moderator analysis suggested that effective techniques could be to encourage self-monitoring, provide face-to-face contact with a facilitator or work on physical activity simultaneously, without providing feedback on behavior, use prompts and cues or provide information about emotional consequences of healthy eating. Table 1 includes examples of each within the studies. These could substantially increase effectiveness: healthy eating interventions with self-monitoring had an SMD of 0.48 compared with the overall SMD of 0.22. Meta-CART analyses exploring combinations tended to confirm that interventions were more effective without feedback, especially when combined with self-monitoring, yet suggested that for interventions which did include feedback, then effects could be stronger when combined with face-to-face contact.

In individual moderator analyses, physical activity interventions tended to be more effective where they had a sole focus on participants being active or were delivered at home or in a community rather than health setting. Activity interventions were more effective where they included direct instruction or opportunities to practice and rehearse active movements but *less* effective when they included information about antecedents (see Table 1, for examples). Meta-CART suggested that a particularly effective combination could be instruction on how to perform the behavior within the community or home (not health) setting.

It may seem surprising that feedback on behavior was associated with lower healthy eating change, despite key behavior change guidance recommending it as a ‘proven technique’ [36]. Yet the two healthy eating interventions with lowest effect sizes [37, 38] provided feedback in a similar, non face-to-face way, through mailed written statements of previously reported healthy eating behavior, and meta-CART suggested that face-to-face delivery could mitigate against the lower effect size. In this review, mailed delivery formats were popular, and e-health technology is increasingly being employed to increase the reach of public health interventions [39] but our review suggested ‘a personal touch’ may be important to support low-income communities in their healthy eating efforts. The finding that self-monitoring without feedback was an effective combination was also surprising given that Control Theory [40] would advocate their combination, and may oppose meta-regression findings that self-monitoring was more effective in healthy eating and physical activity interventions if combined with another Control Theory technique [41]. Again this may be explained by other factors associated with studies where feedback was included.

The strong effect of instruction on physical activity behavior has been found in previous research, [42] although this study adds that delivery of this BCT in a community or home rather than health setting is desirable. Thus, a behavior should be taught in the context that is likely to be (a) easy for participants to attend and (b) as similar as possible to real life to facilitate further performance as associating a behavior with the context is essential to building habits [43]. Additionally, recent evidence suggests that habit mediates the effects of planning on behaivour change [44].

It may also seem counter-intuitive that interventions with a multi-behavior focus (targeting both healthy eating and physical activity) led to increased changes in healthy eating, but that in physical activity a single focus was preferable. Amongst several explanations, it could be assumed that most participants taking part in a multi-component intervention were aiming for weight loss. Since initial weight loss is more easily achieved by calorie restriction than by increased burning through exercise [45], healthy eating may have been the core focus for both intervention facilitators and participants in these studies.

Another finding in this review was that the inclusion of information-focussed BCTs often used in public health interventions such as *4.2 Information about antecedents* for physical activity and *5.6 Information about emotional consequences* for healthy eating resulted in a less successful outcome. This builds on similar findings in different populations [46], further evidence that information (particularly when directed at fear-arousing consequences) is likely to be ineffective without additional BCTs aimed at increasing self-efficacy or planning [18]. A further possibility is that information-giving may dwarf the other more effective components; in a meta-analysis of combinations of components of internet-based interventions for the general public, van Genugten and colleagues [47] found that interventions that were quick to deliver and easy to understand were more effective.

No BCTs or delivery/context variables were associated with smoking intervention effectiveness, in contrast to reviews with pregnant women and people with lung disease respectively which found positive effects for action planning amongst other BCTs [48, 49]. This may reflect the lower heterogeneity in smoking compared to healthy eating and physical activity effects in this review, study authors including a limited range of BCTs in interventions or perhaps poor intervention description [50]. We also found no association between theory use and effectiveness in this review, contrary to some reviews of behavioral interventions [51, 52] but in line with recent diabetes intervention analyses [24, 53]. Similar to other reviews, employing a greater number of techniques was also not linked with increased effectiveness [11, 51].

Many BCTs and delivery/context components could not be analysed as they were seemingly rarely used (e.g. BCT *7.1: Prompts and cues*, identified in one physical activity intervention) or used in all interventions: this could reflect poor reporting of behavioral intervention content [54, 55]. Indeed in the smoking interventions, only 11 BCTs could be analysed. The smoking cessation field may be more extensively developed than others and so perhaps greater consensus has been reached on necessary components of stop smoking support [56].

## Strengths and Limitations

Our review is the first to examine BCTs and delivery/context components using BCTv1 and TIDieR individually and in combination in interventions in low-income adults. The original review identified small positive effects. These new analyses show that larger effects are possible which even exceed overall effects seen in reviews of healthy eating and physical activity interventions for adults in the general population [41, 57, 58], albeit the same interventions are not tested in different population groups.

However, the review has limitations [12], including that the majority of participants were American women, potentially limiting generalizability to other contexts and populations. Primary outcomes varied in the studies: it may be that effective BCTs vary for, for example, increasing fruit consumption compared to reducing dietary fat. As noted in the previous review [12], intervention descriptions were not always detailed, so further BCTs may have been present which could not be coded. Furthermore, poor control group content descriptions prevented their coding altogether and control intervention content may have an important impact on intervention outcomes [59]. In general, this study using meta-CART was exploratory: further work on combinations is needed and though the moderators were chosen as important aspects of content, delivery and context in line with recent behavioral medicine developments, other moderating factors may be important. While the findings on combinations of BCTs and delivery/context components are complex they represent one of the first attempts to investigate these complexities. In practice, BCTs will always be presented in combination with delivery/context components and it is therefore important that these combinations are explored. Future studies are likely to have more evidence to synthesise and may clarify, confirm or suggest that the current results resulted from the relatively small number of studies that allowed these combinations to be investigated. Comparable analyses of interventions with other groups such as individuals with higher incomes would help build the evidence on effective intervention components and elucidate how much tailoring is needed for different population groups.

## Conclusions

This study examined BCTs and delivery/context components associated with effectiveness in healthy eating, physical activity and smoking interventions with a large combined sample of low-income adults. We found that including certain BCTs or delivery/context, individually or in combination, may double a healthy eating or physical activity intervention’s effect size. This adds to the behavior change evidence base and could help public health researchers, policy makers and interventionists increase the effectiveness of healthy eating and physical activity behavior change interventions for disadvantaged, low-income groups.

## Electronic Supplementary Material

Supplementary table 1 (DOCX 33 kb)

Supplementary table 2 (DOCX 22 kb)

Supplementary table 3 (DOCX 19 kb)
