# Supplementary material for: Interventions to Promote Healthy Eating, Physical Activity and Smoking in Low-Income Groups: a Systematic Review with Meta-Analysis of Behavior Change Techniques and Delivery/Context
Source: Int J Behav Med. 2018 Jul 12;25(6):605–16. doi: 10.1007/s12529-018-9734-z (PMC6244564; doi:10.1007/s12529-018-9734-z)
Supplement: Supplementary file 1 — (DOCX 33 kb) [file 12529_2018_9734_MOESM1_ESM.docx]

| **Table 1, Electronic Supplementary File 1: BCT and Categorical Delivery/Context Components Moderator Analyses for Healthy Eating Interventions** | | | | | | | | | | | | | | | | | | |
| --- | --- | --- | --- | --- | --- | --- | --- | --- | --- | --- | --- | --- | --- | --- | --- | --- | --- | --- |
| Healthy eating interventions (k=16) | Meta-analysis (random effects model) | | | | | | | | | | | | | | | Subgroup analysis | | |
| 16 BCTs and 7 delivery/context components coded in between 3 and 13 interventions | Interventions with (without) this variable | $\bar{g}$ BCT/ component present | | 95% CI Lower limit | | 95% CI Upper limit | | $\bar{g}$BCT/ component absent | | | 95% CI Lower limit | | 95% CI Upper limit | | | Q | P | |
| BCTs^15^ | | | | | | | | | | | | | | | | | | |
| 1.1 Goal setting (behavior) | 9 (7) | 0.26 | | 0.15 | | 0.37 | | 0.16 | | | 0.05 | | 0.27 | | | 1.66 | | 0.20 |
| 1.2 Problem solving | 8 (8) | 0.30 | | 0.16 | | 0.43 | | 0.16 | | | 0.07 | | 0.25 | | | 2.78 | | 0.10 |
| 1.4 Action planning | 4 (12) | 0.24 | | 0.07 | | 0.41 | | 0.21 | | | 0.12 | | 0.30 | | | 0.08 | | 0.78 |
| **2.2 Feedback on behavior** | **8 (8)** | **0.14** | | **0.08** | | **0.21** | | **0.36** | | | **0.25** | | **0.48** | | | **11.31** | | **0.00** |
| **2.3 Self-monitoring of behavior** | **4 (12)** | **0.48** | | **0.23** | | **0.74** | | **0.18** | | | **0.11** | | **0.24** | | | **5.27** | | **0.02** |
| 3.1 Social support (unspecified) | 11 (5) | 0.27 | | 0.15 | | 0.39 | | 0.17 | | | 0.10 | | 0.24 | | | 1.94 | | 0.16 |
| 3.2 Social support (practical) | 3 (13) | 0.40 | | 0.11 | | 0.70 | | 0.17 | | | 0.10 | | 0.24 | | | 2.31 | | 0.13 |
| 3.3 Social support (emotional) | 3 (13) | 0.19 | | 0.12 | | 0.26 | | 0.23 | | | 0.12 | | 0.34 | | | 0.47 | | 0.49 |
| 4.1 Instruction on how to perform the behavior | 7 (9) | 0.21 | | 0.06 | | 0.36 | | 0.22 | | | 0.15 | | 0.29 | | | 0.02 | | 0.88 |
| 5.1 Information about health consequences | 6 (16) | 0.23 | | 0.05 | | 0.42 | | 0.21 | | | 0.15 | | 0.26 | | | 0.07 | | 0.79 |
| 5.3 Information on social and environmental consequences | 4 (12) | 0.12 | | -0.02 | | 0.25 | | 0.25 | | | 0.16 | | 0.34 | | | 2.58 | | 0.11 |
| **5.6 Information about emotional consequences** | **3 (13)** | **0.09** | | **-0.03** | | **0.21** | | **0.26** | | | **0.17** | | **0.34** | | | **4.96** | | **0.03** |
| 6.1 Demonstration of the behavior | 5 (11) | 0.36 | | 0.13 | | 0.59 | | 0.18 | | | 0.11 | | 0.25 | | | 2.20 | | 0.14 |
| **7.1 Prompts and cues** | **4 (12)** | **0.09** | | **-0.01** | | **0.19** | | **0.27** | | | **0.18** | | **0.36** | | | **7.06** | | **0.01** |
| 9.1 Credible source | 6 (10) | 0.23 | | 0.06 | | 0.39 | | 0.22 | | | 0.16 | | 0.29 | | | 0.00 | | 0.98 |
| 12.5 Adding objects to the environment | 6 (10) | 0.25 | | 0.06 | | 0.45 | | 0.21 | | | 0.15 | | 0.27 | | | 0.20 | | 0.66 |
| Context/delivery components^14^ | | | | | | | | | | | | | | | | | | |
| WHY: Theoretical base described Yes (no) | 13 (3) | 0.23 | | 0.15 | | 0.32 | | 0.12 | | | -0.04 | | | 0.29 | | 1.37 | | 0.24 |
| HOW: Personal contact included Yes (no) | 10 (6) | 0.27 | | 0.18 | | 0.36 | | 0.13 | | | 0.00 | | | 0.25 | | 3.28 | | 0.07 |
| **HOW: Face-to-face component included Yes (no)** | **8 (8)** | **0.30** | | **0.20** | | **0.40** | | **0.14** | | | **0.04** | | | **0.23** | | **5.04** | | **0.02** |
| Outcome measurement: Self-reported measure only (or more objective measure reported) | 13 (3) | 0.22 | | 0.13 | | 0.31 | | 0.22 | | | 0.08 | | | 0.37 | | 0.01 | | 0.93 |
| WHO RECEIVED: Mixed sex (or all women) | 7 (9) | 0.16 | | 0.09 | | 0.23 | | 0.30 | | | 0.16 | | | 0.45 | | 3.09 | | 0.08 |
| **Number of behaviors targeted: one (or more than one)** | **10 (6)** | **0.14** | | **0.08** | | **0.20** | | **0.39** | | | **0.21** | | | **0.56** | | **6.84** | | **0.01** |
|  | | SMD Community Setting | 95% CI Lower limit | | 95% CI Upper limit | | SMD Health setting | | 95% CI Lower limit | 95% CI Upper limit | | SMD Home Setting | | 95% CI Lower limit | 95% CI Upper limit | Q | P | |
| WHERE: Study setting community, health or home | 2, 6, 8 | 0.26 | 0.08 | | 0.45 | | 0.24 | | 0.17 | 0.31 | | 0.199 | | 0.06 | 0.33 | 0.48 | 0.79 | |

**Bold type** = statistically significant difference in subgroups *p<.05* for this variable
