# Supplementary material for: Interventions to Promote Healthy Eating, Physical Activity and Smoking in Low-Income Groups: a Systematic Review with Meta-Analysis of Behavior Change Techniques and Delivery/Context
Source: Int J Behav Med. 2018 Jul 12;25(6):605–16. doi: 10.1007/s12529-018-9734-z (PMC6244564; doi:10.1007/s12529-018-9734-z)
Supplement: Supplementary file 2 — (DOCX 22 kb) [file 12529_2018_9734_MOESM2_ESM.docx]

|  |  | **Table 2: BCT and Categorical Delivery/Context Components Moderator Analyses for Physical Activity Interventions** | | | | | | | | | | | | |
| --- | --- | --- | --- | --- | --- | --- | --- | --- | --- | --- | --- | --- | --- | --- |
|  |  | Physical Activity interventions (k=12) | Meta-analysis (random effects model) | | | | | | | | | Subgroup analysis | | |
|  |  | 14 BCTs and 6 delivery/context components coded in between 3 and 9 physical activity interventions | Interventions with (without) this variable | $\bar{g}$ BCT/ component  Present | | 95% CI Lower limit | 95% CI Upper limit | $\bar{g}$ BCT/ component  Absent | 95% CI Lower limit | | 95% CI Upper limit | Q | | P |
|  |  | BCTs^15^ | | | | | | | | | | | | |
|  |  | 1.1 Goal setting (behavior) | 7 (5) | 0.19 | | -0.02 | 0.40 | 0.26 | 0.10 | | 0.41 | 0.27 | | 0.60 |
|  |  | 1.2 Problem solving | 9 (3) | 0.19 | | 0.01 | 0.36 | 0.26 | 0.04 | | 0.47 | 0.25 | | 0.61 |
|  |  | 1.4 Action planning | 4 (8) | 0.08 | | -0.10 | 0.27 | 0.26 | 0.06 | | 0.46 | 1.60 | | 0.21 |
|  |  | 2.2 Feedback on behavior | 3 (9) | 0.31 | | -0.13 | 0.74 | 0.21 | 0.08 | | 0.34 | 0.18 | | 0.68 |
|  |  | 2.3 Self-monitoring of behavior | 5 (7) | 0.22 | | 0.01 | 0.43 | 0.21 | 0.00 | | 0.41 | 0.00 | | 0.96 |
|  |  | 3.2 Social support (practical) | 4 (8) | 0.12 | | -0.12 | 0.36 | 0.29 | 0.17 | | 0.41 | 1.49 | | 0.22 |
|  |  | **4.1 Instruction on how to perform the behavior** | **5 (7)** | **0.35** | | **0.20** | **0.51** | **0.02** | **-0.05** | | **0.10** | **14.18** | | **0.00** |
|  |  | **4.2 Information about antecedents** | **3 (9)** | **-0.01** | | **-0.10** | **0.07** | **0.26** | **0.12** | | **0.39** | **11.02** | | **0.00** |
|  |  | 5.1 Information about health consequences | 3 (9) | 0.19 | | -0.02 | 0.40 | 0.20 | 0.01 | | 0.39 | 0.01 | | 0.91 |
|  |  | 5.3 Information on social and environmental consequences | 3 (9) | 0.26 | | -0.03 | 0.56 | 0.19 | 0.01 | | 0.37 | 0.16 | | 0.69 |
|  |  | 6.1 Demonstration of the behavior | 4 (6) | 0.29 | | 0.14 | 0.44 | 0.14 | -0.05 | | 0.33 | 1.48 | | 0.22 |
|  |  | **8.1 Behavioral practice/ rehearsal** | **5 (7)** | **0.35** | | **0.20** | **0.51** | **0.02** | **-0.06** | | **0.09** | **14.37** | | **0.00** |
|  |  | 9.1 Credible source | 3 (9) | 0.35 | | 0.02 | 0.67 | 0.17 | -0.01 | | 0.34 | 0.92 | | 0.34 |
|  |  | 10.9 Self-reward | 4 (8) | 0.33 | | 0.04 | 0.63 | 0.17 | -0.01 | | 0.34 | 0.87 | | 0.35 |
|  |  | Context/delivery components^14^ | | | | | | | | | | | | |
|  |  | HOW: Personal contact included Yes (no) | 7 (5) | 0.19 | | -0.03 | 0.41 | 0.24 | 0.08 | | 0.40 | 0.13 | | 0.72 |
|  |  | HOW: Face-to-face component included Yes (no) | 6 (6) | 0.19 | | -0.05 | 0.43 | 0.24 | 0.11 | | 0.38 | 0.17 | | 0.68 |
|  |  | Outcome measurement: Self-reported measure only (or more objective measure reported) | 8 (4) | 0.16 | | 0.01 | 0.30 | 0.32 | 0.08 | | 0.56 | 1.27 | | 0.26 |
|  |  | WHO RECEIVED: Mixed sex (or all women) | 4 (8) | 0.18 | | -0.10 | 0.47 | 0.22 | 0.07 | | 0.37 | 0.05 | | 0.82 |
|  |  | **Number of behaviors targeted: one (or more than one)** | **6 (6)** | **0.30** | | **0.16** | **0.45** | **0.09** | **-0.05** | | **0.22** | **4.72** | | **0.03** |
|  |  |  | | SMD Community Setting | 95% CI Lower limit | 95% CI Upper limit | SMD Health setting | 95% CI Lower limit | 95% CI Upper limit | SMD Home Setting | 95% CI Lower limit | 95% CI Upper limit | Q | P |
|  |  | **WHERE: Study setting community, health, or home** | **3, 5, 4** | **0.40** | **0.29** | **0.51** | **0.00** | **-0.07** | **0.08** | **0.33** | **0.17** | **0.5** | **39.17** | **0.00** |

**Bold type** = statistically significant difference in subgroups *p<.05* for this variable
