# Supplementary material for: Interventions to Promote Healthy Eating, Physical Activity and Smoking in Low-Income Groups: a Systematic Review with Meta-Analysis of Behavior Change Techniques and Delivery/Context
Source: Int J Behav Med. 2018 Jul 12;25(6):605–16. doi: 10.1007/s12529-018-9734-z (PMC6244564; doi:10.1007/s12529-018-9734-z)
Supplement: Supplementary file 3 — (DOCX 19 kb) [file 12529_2018_9734_MOESM3_ESM.docx]

| **Table 3 BCT and Categorical Delivery/Context Components Moderator Analyses for Smoking Interventions** | | | | | | | | | | | | | | | | | | | | | | | | | | | | | | | | | | | | | | | | |
| --- | --- | --- | --- | --- | --- | --- | --- | --- | --- | --- | --- | --- | --- | --- | --- | --- | --- | --- | --- | --- | --- | --- | --- | --- | --- | --- | --- | --- | --- | --- | --- | --- | --- | --- | --- | --- | --- | --- | --- | --- |
| Smoking interventions (k=17) | Meta-analysis (random effects model) | | | | | | | | | | | | | | | | | | | | | | | | | | | | | | | | | | | | Subgroup analysis | | | |
| 11 BCTs and 5 delivery/context components coded in between 3 and 14 interventions | Interventions with (without) this variable | | | RR BCT/component Present | | 95% CI Lower limit | | | Upper limit | | | | | Z  (P) | | | RR BCT/component Absent | | | | | | | 95% CI Lower limit | | | | Upper limit | | | | | | Z  (P) | | | Q | | | P |
| BCTs^15^ | | | | | | | | | | | | | | | | | | | | | | | | | | | | | | | | | | | | | | | | |
| 1.1 Goal setting (behavior) | 4 (13) | | | 1.84 | | 0.93 | | | 3.67 | | | 1.74 (0.08) | | | | | 1.56 | | | | | 1.31 | | | | | 1.86 | | | | | | 4.95 (0.00) | | | | 0.21 | | | 0.65 |
| 1.2 Problem solving | 9 (8) | | | 1.69 | | 1.27 | | | 2.26 | | | 3.56 (0.00) | | | | | 1.55 | | | | | 1.22 | | | | | 1.97 | | | | | | 3.55 (0.00) | | | | 0.22 | | | 0.64 |
| 1.4 Action planning | 6 (11) | | | 1.92 | | 1.37 | | | 2.68 | | | 3.80 (0.00) | | | | | 1.48 | | | 1.18 | | | | | | | 1.84 | | | | | | 3.45 (0.00) | | | | 1.62 | | | 0.20 |
| 2.1 Monitoring of behavior by others without feedback | 3 (14) | | | 1.44 | | 0.73 | | | 2.87 | | | 1.05 (0.29) | | | | | 1.63 | | | 1.38 | | | | | | | 1.93 | | | | | | 5.78 (0.00) | | | | 0.12 | | | 0.73 |
| 3.1 Social support (unspecified) | 12 (5) | | | 1.63 | | 1.37 | | | 1.93 | | | 5.57 (0.00) | | | | | 1.44 | | | | | 0.87 | | | | | 2.39 | | | | | | 1.42 (0.16) | | | | 0.20 | | | 0.65 |
| 3.2 Social support (practical) | 5 (12) | | | 1.54 | | 0.94 | | | 2.52 | | | 1.72 (0.08) | | | | | 1.62 | | | | | 1.35 | | | | | 1.94 | | | | | | 5.19 (0.00) | | | | 0.03 | | | 0.85 |
| 3.3 Social support (emotional) | 3 (14) | | | 1.44 | | 0.73 | | | 2.87 | | | 1.05 (0.29) | | | | | 1.63 | | | | | 1.38 | | | | | 1.93 | | | | | | 5.78 (0.00) | | | | 0.12 | | | 0.73 |
| 5.1 Information about health consequences | 5 (12) | | | 1.89 | | 1.33 | | | | 2.69 | | | 3.55 (0.00) | | | | 1.49 | | | 1.23 | | | | | | | 1.81 | | | | | | 4.04 (0.00) | | | | 1.35 | | | 0.25 |
| 10.4 Social reward | 3 (14) | | | 1.45 | | 1.12 | | | | 1.89 | | | 2.80 (0.01) | | | | 1.64 | | | 1.31 | | | | | | | 2.05 | | | | | | 4.39 (0.00) | | | | 0.47 | | | 0.49 |
| 11.1 Pharmacological support | 13 (4) | | | 1.68 | | | 1.39 | | | 2.03 | | | 5.32 (0.00) | | | | 1.35 | | | | 0.90 | | | | | | 2.01 | | | | | | 1.45 (0.15) | | | | 0.96 | | | 0.33 |
| 11.2 Reduce negative emotions | 5 (12) | | | 1.52 | | | 0.99 | | | 2.35 | | | 1.92 (0.06) | | | | 1.63 | | | | 1.36 | | | | | | 1.97 | | | | | | 5.18 (0.00) | | | | 0.08 | | | 0.77 |
| Context/delivery components^14^ | | | | | | | | | | | | | | | | | | | | | | | | | | | | | | | | | | | | | | | | |
| WHY: Theoretical base described Yes (no) | 9 (8) | | | 1.48 | | | 1.23 | | | 1.79 | | | 4.08 (0.00) | | | | 1.80 | | | | | | 1.28 | | | 2.54 | | | | | | 3.36 (0.00) | | | | 0.96 | | | 0.33 | |
| HOW: Face-to-face component included Yes (no) | 13 (4) | | | 1.70 | | | 1.37 | | | 2.13 | | | 4.71 (0.00) | | | | 1.41 | | | | | | 0.99 | | | 2.00 | | | | | | 1.92 (0.05) | | | | 0.81 | | | 0.37 | |
| Outcome measurement: Self-reported measure only (or more objective measure reported) | 7 (10) | | | 1.52 | | | 1.26 | | | 1.83 | | | 4.32 (0.00) | | | | 1.69 | | | | | | 1.24 | | | 2.32 | | | | | | 3.27 (0.00) | | | | 0.35 | | | 0.55 | |
| WHO RECEIVED: Mixed sex (or all women) | 9 (8) | | | 1.74 | | | 1.35 | | | 2.25 | | | 4.31 (0.00) | | | | 1.47 | | | | | | 1.15 | | | 1.89 | | | | | | 3.05 (0.00) | | | | 0.86 | | | 0.35 | |
|  | | | RR Community setting | | 95% CI Lower limit | | | 95% CI Upper limit | | | Z (P) | | | | RR Health setting | 95% CI Lower limit | | 95% CI Upper limit | Z (P) | | | | | | RR Home settting | | | | 95% CI Lower limit | | 95% CI Upper limit | | | | Z (P) | Q | | | P | |
| WHERE: Study setting community, health or home | | 3,6,7 | 1.44 | | 0.55 | | | 3.79 | | | 0.74 (0.46) | | | | 1.98 | 1.37 | | 2.85 | 3.67 (0.00) | | | | | | 1.47 | | | | 1.19 | 1.82 | | | | | 3.56 (0.00) | | 1.94 | 0.38 | | |

**Bold type** = statistically significant difference in subgroups *p<.05* for this variable (no significant differences in Table 3)
